# Potential role of MALT1 as a candidate biomarker of disease surveillance and treatment response prediction in inflammatory bowel disease patients

**DOI:** 10.1002/jcla.24130

**Published:** 2022-01-08

**Authors:** Zhigang Wu, Yingyan Bi

**Affiliations:** ^1^ General Surgery Yulin No. 2 Hospital Yulin Shaanxi China; ^2^ Department of Pharmacy Gansu Province Hospital of Traditional Chinese Medicine Lanzhou Gansu China

**Keywords:** disease risk, inflammatory bowel disease, inflammatory cytokines, mucosa‐associated lymphoid tissue lymphoma translocation protein 1, treatment response

## Abstract

**Background:**

Mucosa‐associated lymphoid tissue lymphoma translocation protein 1 (MALT1) regulates adaptive and innate immune responses in several inflammatory disease. However, clinical involvement of MALT1 in inflammatory bowel disease (IBD) patients remains unclear. Hence, this study was intended to investigate the correlation of blood MALT1 with disease activity, inflammation indexes as well as treatment response of IBD patients.

**Methods:**

Blood MALT1 expression in 100 IBD patients [including 25 active (A)‐Crohn's disease (CD) patients, 25 remission (R)‐CD patients, 25 A‐ulcerative colitis (UC) patients, and 25 R‐UC patients] and 25 health controls (HCs) was detected by reverse transcription‐quantitative polymerase chain reaction; besides, serum tumor necrosis factor‐alpha (TNF‐α) and interleukin‐17A (IL‐17A) in IBD patients were detected by enzyme‐linked immunosorbent assay.

**Results:**

MALT1 was increased in A‐UC patients than in R‐UC patients (*p* = 0.038) and in HCs (*p* < 0.001), and also elevated in A‐CD patients than in R‐CD patients (*p* = 0.048) and in HCs (*p* < 0.001). MALT1 was positively related to C‐reactive protein (CRP, *p* = 0.011), TNF‐α (*p* = 0.036), IL‐17A (*p* = 0.023), and Mayo score (*p* = 0.005) in A‐UC patients, CRP (*p* = 0.017), erythrocyte sedimentation rate (*p* = 0.033), TNF‐α (*p* = 0.004), and Crohn's disease activity index score (*p* = 0.028) in A‐CD patients. But MALT1 was not correlated with either inflammation indexes or disease activity score in R‐UC and R‐CD patients. MALT1 gradually declined from baseline to W12 in A‐UC and A‐CD patients (both *p* < 0.001). Moreover, MALT1 at W4 (*p* = 0.031) and W12 (*p* = 0.003) in A‐UC patients as well as MALT1 at W12 (*p* = 0.008) in A‐CD patients associated with clinical response.

**Conclusion:**

MALT1 serves as a potential biomarker for disease surveillance and treatment response prediction of IBD patients.

## Graphical abstract

A total of 100 inflammatory bowel disease (IBD) [including 25 active (A)‐Crohn's disease (CD) patients, 25 remission (R)‐CD patients, 25 A‐ulcerative colitis (UC) patients, and 25 R‐UC patients] and 25 health controls (HCs) were enrolled to detect their blood mucosa‐associated lymphoid tissue lymphoma translocation protein 1 (MALT1); besides, serum tumor necrosis factor‐alpha (TNF‐α) and interleukin‐17A (IL‐17A) in IBD patients were also evaluated. This study found that MALT1 was increased in A‐UC patients than in R‐UC patients and in HCs, and it also elevated in A‐CD patients than in R‐CD patients and in HCs. Furthermore, MALT1 was positively related to C‐reactive protein (CRP), TNF‐α, IL‐17A, and mayo score in A‐UC patients; positively correlated with CRP, erythrocyte sedimentation rate, TNF‐α, and Crohn's disease activity index score in A‐CD patients. But MALT1 was not correlated with either inflammation indexes or disease activity score in R‐UC and R‐CD patients. Moreover, MALT1 gradually declind from baseline to W12 in A‐UC and A‐CD patients; meanwhile, MALT1 decrement at W4 and W12 in A‐UC patients as well as MALT1 at W12 in A‐CD patients associated with clinical response.

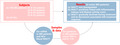

## 1 INTRODUCTION

Inflammatory bowel disease (IBD), characterized by continuing aberrant immune response, is an idiopathic gut‐inflammatory disease with an increasing incidence, which predominantly includes ulcerative colitis (UC) and Crohn's disease (CD).^1^, ^2^, ^3^, ^4^, ^5^ In order to alleviate clinical symptoms (including abdominal pain, watery, or bloody diarrhea, etc.), promote intestinal mucosa healing, and reduce disease activity, many medical treatments have been applied in IBD patients, including steroids, immunosuppressants, biological treatments, etc.^6^, ^7^ However, IBD is incurable and easy to relapse, which brings treatment‐cost burden to patients and their families; moreover, reliable methods to monitor disease progression in IBD patients are still lacking.^8^, ^9^, ^10^, ^11^ Therefore, it is necessary to explore novel biomarkers to estimate disease risk and activity as well as treatment response of IBD; subsequently provide individual treatment and improve outcomes of IBD patients.

Mucosa‐associated lymphoid tissue lymphoma translocation protein 1 (MALT1) is located close to B‐cell lymphoma (BCL) 2 on chromosome 18q21 and expressed in multiple cell types.^12^, ^13^, ^14^ Based on its scaffold function and proteolytic activity, MALT1 regulates immune reactions via activating several signaling pathways (mainly including nuclear factor‐kappa‐B (NF‐κB) signaling pathway, etc.).^15^, ^16^ Moreover, MALT1 induces the differentiation of T‐helper (Th) 1 and Th17 cells, which further aggravates many inflammatory and autoimmune diseases, such as psoriasis, rheumatoid arthritis, etc.^17^, ^18^, ^19^ Besides, as mentioned above, IBD is a chronic inflammatory disease, which is characterized by abnormal intestinal immune response.^4^ Therefore, we speculated that MALT1 might be involved in the etiology and development of IBD. However, there is no clinical research noting that MALT1 can serve as a potential biomarker for evaluation of disease risk and activity of IBD.

Hence, this study detected blood MALT1 expression in IBD patients at baseline, week (W) 4 and W12 after treatment, aiming to investigate the correlation of MALT1 with disease activity, inflammation indexes, and treatment response in IBD patients.

## 2 METHODS

### 2.1 Subjects

This study consecutively enrolled a total of 100 IBD patients who were treated in our hospital between January 2019 and May 2021, including 25 active CD patients (A‐CD), 25 CD patients in clinical remission (R‐CD), 25 active UC patients (A‐UC), and 25 UC patients in clinical remission (R‐UC). The patients were included if they had the following conditions: (a) diagnosis of IBD (including CD and UC) according to Chinese consensus on diagnosis and treatment in IBD^20^; (b) aged over 18 years; (c) willing to provide peripheral blood (PB) for study use. Besides, A‐CD patients were required to have a Crohn's disease activity index (CDAI) score ≥150 points, and R‐CD patients were required to have a CDAI score <150 points; A‐UC patients were required to have a Mayo score ≥3 points, and R‐UC patients were required to have a Mayo score <3 points.^21^ The patients were excluded from the study if they had other immune system diseases, severe active infections, hematologic malignancies, or cancers. In addition, another 25 healthy subjects with matched gender and age to IBD patients were recruited as health controls (HCs), who were also excluded if they had immune system diseases, severe active infections, hematologic malignancies, or cancers. The study was approved by Institutional Research Ethics Committee of Gansu Province Hospital of Traditional Chinese Medicine.

### 2.2 Collection of data and samples

Characteristics of all subjects were recorded after enrollment and examination, which included age, gender, C‐reactive protein (CRP), and erythrocyte sedimentation rate (ESR). Besides, CDAI score was collected from CD patients, and Mayo score was collected from UC patients. The higher CDAI score and Mayo score indicated the more severe disease activity. For sample collection, PB samples of all subjects were obtained after enrollment to separate peripheral blood mononuclear cells (PBMCs) and serum. Besides, for A‐CD patients and A‐UC patients, PB samples were also obtained at W4 and W12 to isolate PBMCs.

### 2.3 Assessment of inflammatory cytokine level

Inflammatory cytokines in serum of all IBD patients, including tumor necrosis factor‐alpha (TNF‐α) and interleukin‐17A (IL‐17A), were detected by enzyme‐linked immunosorbent assay (ELISA) using commercial Human ELISA Kits (Bio‐Techne China Co., Ltd.). The ELISA procedure was strictly followed the instructions of kits.

### 2.4 Assessment of MALT1 expression

MALT1 expression in PBMCs was detected by reverse transcription‐quantitative polymerase chain reaction (RT‐qPCR) assay among all subjects after enrollment, as well as A‐CD patients and A‐UC patients at W4 and W12 after treatment. In brief, total RNA was extracted using RNeasy Protect Mini Kit (Qiagen). Then reserve transcription was completed by PrimeScript™ RT reagent Kit (Takara). After that, qPCR reaction was finished using SYBR^®^ Premix DimerEraser™ (Takara). The relative expression was calculated by 2^−ΔΔCt^ method using GAPDH as the internal reference. The primer sequences were referred to the previous study.^22^

### 2.5 Assessment of clinical response

At W12 after treatment, clinical response was assessed among A‐CD patients and A‐UC patients. Clinical response of A‐CD patients was defined as a CDAI score decrease over 70 points from baseline^21^; clinical response of A‐UC patients was defined as a Mayo score decrease over 30% or 3 points from baseline, and the rectal bleeding subscore decrease at least 1 point or the rectal bleeding subscore of 0–1.^23^

### 2.6 Statistical analysis

Statistical analysis and graph plotting were carried out using SPSS 24.0 software (IBM Corp.) and GraphPad Prism 6.01 software (GraphPad Software, Inc.), respectively. Comparisons of baseline characteristics among groups were determined by one‐way analysis of variance test, Kruskal‐Wallis H rank sum test, Wilcoxon rank sum test, and Chi‐square test. MALT1 expression among groups was compared using Kruskal‐Wallis H rank sum test and adjusted using Bonferroni method. The ability of MALT1 expression in distinguishing CD patients, UC patients, and HCs was evaluated by receiver‐operating characteristic analysis. Correlations between MALT1 expression and clinical characteristics were analyzed by Spearman's rank correlation test. Changes of MALT1 expression over time were evaluated using Friedman test. MALT1 expression between patients with or without clinical response at W12 was compared using Wilcoxon rank‐sum test. Statistical significance was concluded if a two‐side *p* value less than 0.05.

## 3 RESULTS

### 3.1 Characteristics of IBD patients and HCs

The mean ages of A‐CD, R‐CD, A‐UC, R‐UC, and HCs were 31.6 ± 9.8 years, 38.1 ± 10.6 years, 36.2 ±11.8 years, 38.1 ± 11.0 years, and 36.2 ± 10.4 years, respectively (*p* = 0.205, Table 1). There were 14 (56.0%) males and 11 (44.0%) females in A‐CD group, 9 (36.0%) males and 16 (64.0%) females in R‐CD group, 13 (52.0%) males and 12 (48.0%) females in A‐UC group, 11 (44.0%) males and 14 (56.0%) females in R‐UC group, and 12 (48.0%) males and 13 (52%) females in HCs (*p* = 0.667). Besides, CRP and ESR were highest in active patients, then remission patients and lowest in HCs (both *p* < 0.001). Additionally, TNF‐α was increased in active patients than in remission patients (*p* = 0.017). The detailed characteristics are listed in Table 1.

**TABLE 1:** Characteristics of IBD patients and HCs

Items	HCs (N = 25)	R‐UC (N = 25)	A‐UC (N = 25)	R‐CD (N =25)	A‐CD (N = 25)	Statistic (F/χ^2^/Z/H)	*p* value
Age (years), mean ± SD	36.2 ± 10.4	38.1 ± 11.0	36.2 ± 11.8	38.1 ± 10.6	31.6 ± 9.8	1.503	0.205
Gender, No. (%)						2.375	0.667
Male	12 (48.0)	11 (44.0)	13 (52.0)	9 (36.0)	14 (56.0)		
Female	13 (52.0)	14 (56.0)	12 (48.0)	16 (64.0)	11 (44.0)		
CRP (mg/L), median (IQR)	2.6 (2.1–5.0)	17.1 (12.6–25.5)	69.3 (45.2–88.1)	16.1 (12.4–27.2)	56.7 (39.4–85.4)	96.717	<0.001
ESR (mm/h), median (IQR)	10.5 (7.3–13.5)	14.6 (10.6–21.4)	45.9 (34.8–63.0)	18.4 (13.4–30.8)	41.5 (33.7–61.9)	82.683	<0.001
TNF‐α (pg/ml), median (IQR)	‐	87.9 (47.7–124.1)	144.8 (95.4–199.4)	65.0 (47.1–123.7)	136.1 (60.1–204.2)	10.239	0.017
IL−17A (pg/ml), median (IQR)	‐	77.1 (50.0–161.9)	104.4 (68.5–198.6)	59.2 (49.0–129.8)	95.0 (72.6–202.0)	5.134	0.162
CDAI, median (IQR)	‐	‐	‐	118.0 (87.5–127.0)	264.0 (210.0–323.5)	−6.064	<0.001
Mayo score, median (IQR)	‐	1.0 (1.0–2.0)	6.0 (5.0–8.0)	‐	‐	−6.163	<0.001

Abbreviations: A‐CD, active Crohn's disease; A‐UC, active ulcerative colitis; CDAI, Crohn's disease activity index; CRP, C‐reactive protein; ESR, erythrocyte sedimentation rate; HCs, health controls; IBD, inflammatory bowel disease; IL‐17A, interleukin 17A; IQR, interquartile range; R‐CD, Crohn's disease in remission; R‐UC, ulcerative colitis in remission; SD, standard deviation; TNF‐α, tumor necrosis factor alpha.

### 3.2 Expression of MALT1 in IBD patients and HCs

**FIGURE 1:**
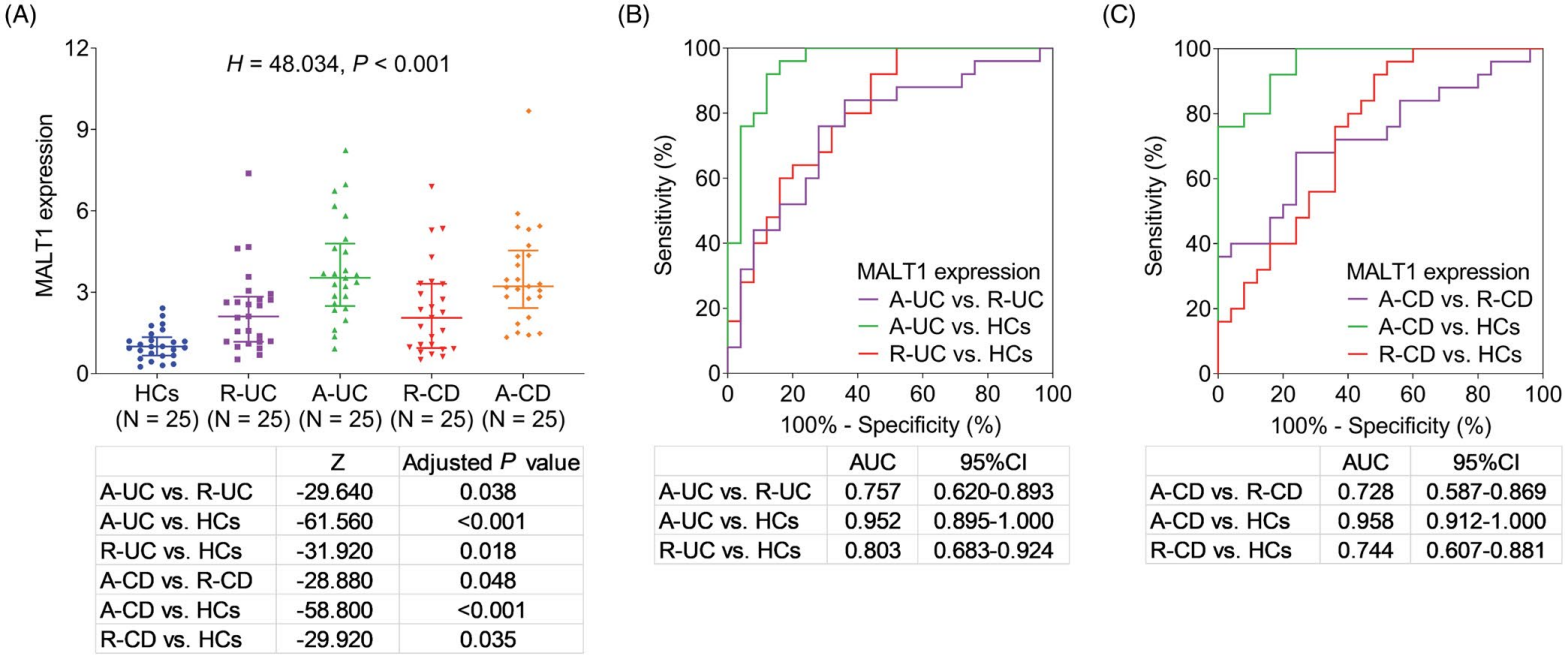
MALT1 expression in all subjects. The expression of MALT1 in A‐CD patients, R‐CD patients, A‐UC patients, R‐UC patients, and HCs (A) and its diagnostic value in distinguishing UC patients versus HCs and A‐UC patients versus R‐UC patients (B), CD patients versus HCs and A‐CD patients versus R‐CD patients (C). AUC, area under curve; CD, Crohn's disease; CI, confidence interval; HCs, health controls; MALT1, mucosa‐associated lymphoid tissue lymphoma translocation protein 1; UC, ulcerative colitis

Generally, difference was observed in MALT1 among A‐CD patients, R‐CD patients, A‐UC patients, R‐UC patients, and HCs (*p* < 0.001, Figure 1A). In detail: MALT1 in both A‐UC patients and A‐CD patients was elevated than that in HCs (both *p* < 0.001); besides, MALT1 was elevated in A‐UC patients versus R‐UC patients (*p* = 0.038) and A‐CD patients versus R‐CD patients (*p* = 0.048).

Moreover, MALT1 disclosed good value to distinguish A‐UC patients from HCs (area under curve [AUC]: 0.952, 95% confidence interval [CI]: 0.895–1.000) and A‐UC patients from R‐UC patients (AUC: 0.757, 95% CI: 0.620–0.893) (Figure 1B). Meanwhile, MALT1 also had good value to distinguish A‐CD patients from HCs (AUC: 0.958, 95% CI: 0.912–1.000) as well as A‐CD patients from R‐CD patients (AUC: 0.728, 95% CI: 0.587–0.869) (Figure 1C).

### 3.3 Correlation of MALT1 with disease activity score in IBD patients

**FIGURE 2:**
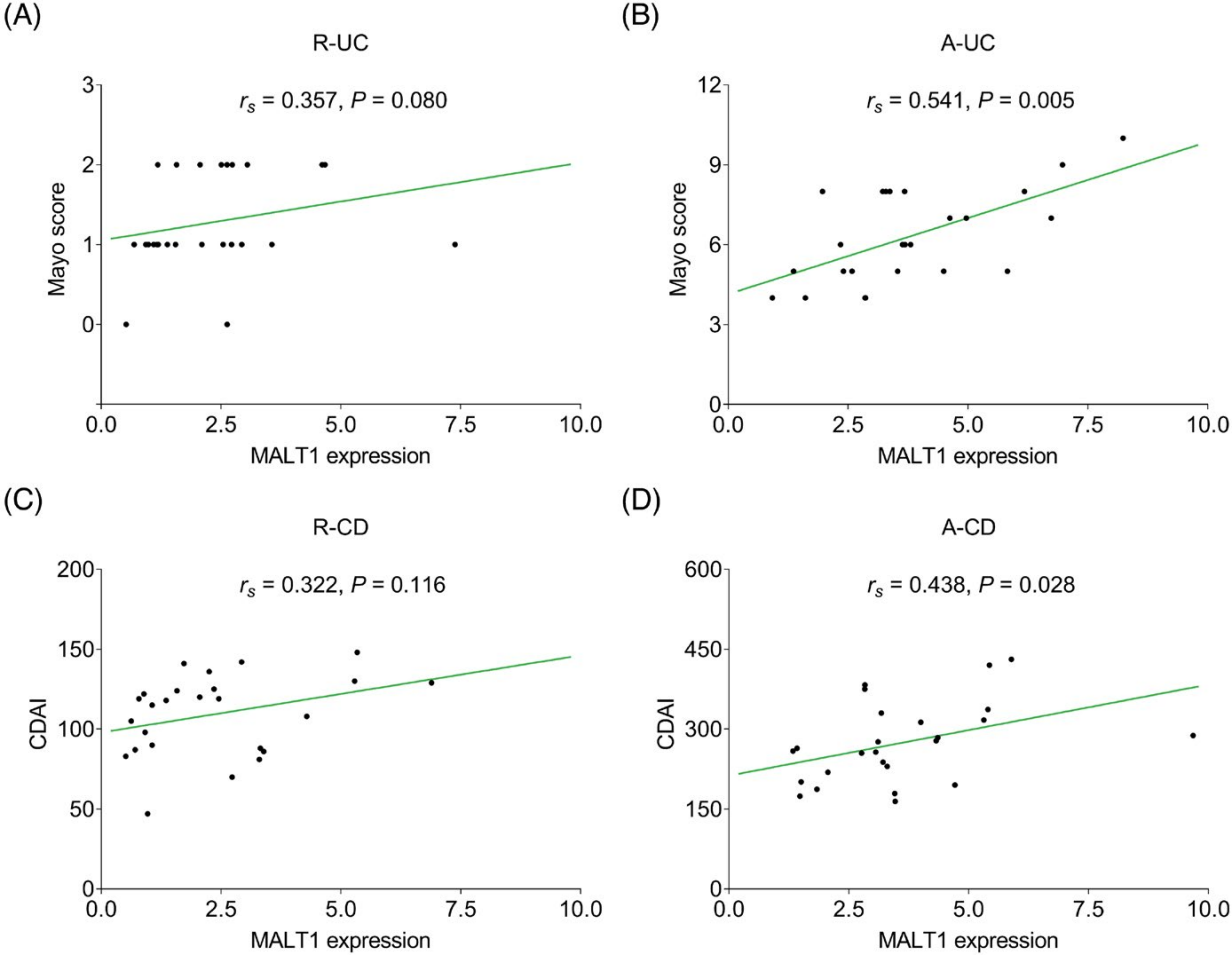
MALT1 positively correlated with mayo score and CDAI score in active IBD patients. The correlation of MALT1 with mayo score in R‐UC (A) and A‐UC (B) patients; the correlation of MALT1 with CDAI score in R‐CD (C) and A‐CD (D) patients. A‐CD, active Crohn's disease patients; A‐UC, active ulcerative colitis patients; CDAI, Crohn's disease activity index; MALT1, mucosa‐associated lymphoid tissue lymphoma translocation protein 1; R‐CD, Crohn's disease in clinical remission; R‐UC, ulcerative colitis patients in clinical remission

MALT1 was positively correlated with mayo score in A‐UC patients (*r* = 0.541, *p* = 0.005), but they were not correlated in R‐UC patients (*r* = 0.357, *p* = 0.080) (Figure 2A,B). In terms of CD, MALT1 was positively associated with CDAI score in A‐CD patients (*r* = 0.438, *p* = 0.028), while there was no association between MALT1 and CDAI score in R‐CD patients (*r* = 0.322, *p* = 0.116) (Figure 2C,D).

### 3.4 Correlation of MALT1 with inflammation indexes in IBD patients

MALT1 was positively correlated with CRP (*r* = 0.499, *p* = 0.011), TNF‐α (*r* = 0.422, *p* = 0.036), and IL‐17A (*r* = 0.452, *p* = 0.023) in A‐UC patients. Furthermore, MALT1 was positively linked with CRP (*r* = 0.475, *p* = 0.017), ESR (*r* = 0.428, *p* = 0.033), and TNF‐α (*r* = 0.552, *p* = 0.004) in A‐CD patients. No association was found between MALT1 and inflammation indexes in R‐UC and R‐CD patients (all *p* > 0.05) (Table 2).

**TABLE 2:** Correlation of MALT1 expression with inflammation indexes in IBD patients

Items	MALT1 expression in R‐UC		MALT1 expression in A‐UC		MALT1 expression in R‐CD		MALT1 expression in A‐CD	
	*r*_s_	*p* value	*r*_s_	*p* value	*r*_s_	*p* value	*r*_s_	*p* value
CRP	0.336	0.101	0.499	0.011	0.286	0.166	0.475	0.017
ESR	0.092	0.663	0.305	0.138	0.273	0.187	0.428	0.033
TNF‐α	0.280	0.175	0.422	0.036	0.242	0.243	0.552	0.004
IL−17A	0.248	0.233	0.452	0.023	0.195	0.349	0.320	0.119

Abbreviations: A‐CD, active Crohn's disease; A‐UC, active ulcerative colitis; CRP, C‐reactive protein; ESR, erythrocyte sedimentation rate; IBD, inflammatory bowel disease; IL‐17A, interleukin 17A; MALT1, mucosa‐associated lymphoid tissue lymphoma translocation protein 1; R‐CD, Crohn's disease in remission; R‐UC, ulcerative colitis in remission; TNF‐α, tumor necrosis factor alpha.

### 3.5 Comparison of MALT1 at different timepoints after treatment in active IBD patients

**FIGURE 3:**
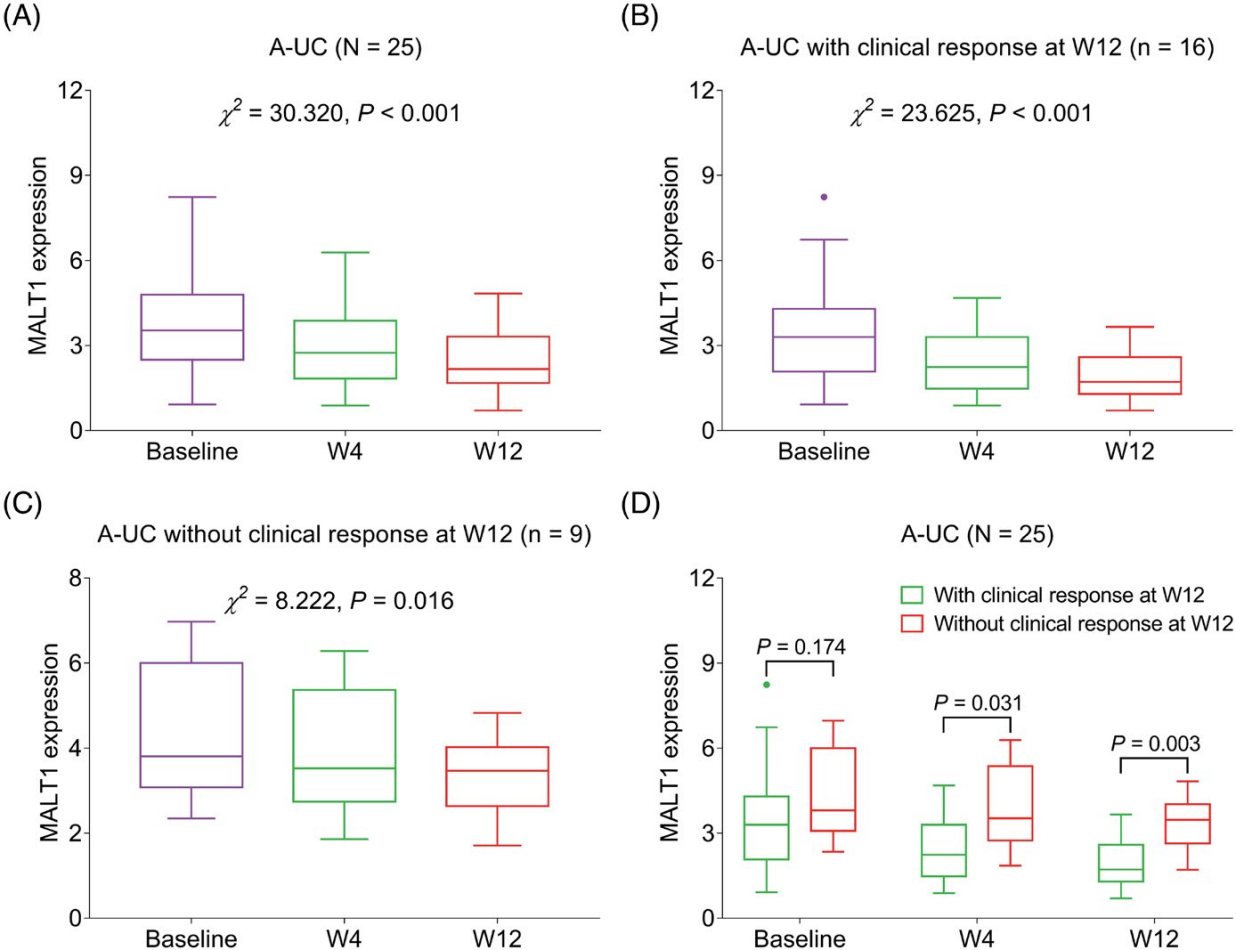
MALT1 was gradually declined during 12‐week treatment in A‐UC patients and correlated with treatment response. Comparison of MALT1 from baseline to W12 after treatment in total A‐UC patients (A), A‐UC patients with clinical response (B), and A‐UC patients without clinical response (C); comparison of MALT1 at baseline, W4 and W12 between patients with and without clinical response (D). A‐UC, active ulcerative colitis patients; MALT1, mucosa‐associated lymphoid tissue lymphoma translocation protein 1; W, week

MALT1 was gradually declined from baseline to W12 in total A‐UC patients (*p* < 0.001), A‐UC patients with clinical response (*p* < 0.001), and A‐UC patients without clinical response (*p* = 0.016) (Figure 3A–C). Moreover, there was no difference of MALT1 at baseline between A‐UC patients with and without clinical response (*p* = 0.174), while MALT1 at W4 (*p* = 0.031) and W12 (*p* = 0.003) was lower in patients with clinical response compared to those without clinical response (Figure 3D).

**FIGURE 4:**
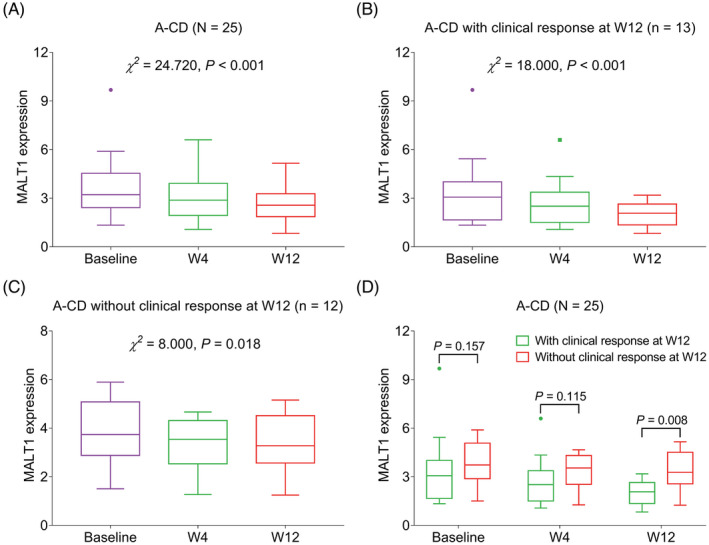
MALT1 was gradually declined during 12‐week treatment in A‐CD patients and correlated with treatment response. Comparison of MALT1 from baseline to W12 after treatment in total A‐CD patients (A), A‐CD patients with clinical response (B), and A‐CD patients without clinical response (C); comparison of MALT1 at baseline, W4 and W12 between patients with and without clinical response (D). A‐CD, active Crohn's disease patients; MALT1, mucosa‐associated lymphoid tissue lymphoma translocation protein 1; W, week

In terms of A‐CD patients, MALT1 was also gradually decreased from baseline to W12 in total A‐CD patients (*p* < 0.001), A‐CD patients with clinical response (*p* < 0.001), and A‐CD patients without clinical response (*p* = 0.018) (Figure 4A–C). Furthermore, no difference of MALT1 at baseline (*p* = 0.157) or W4 (*p* = 0.115) was observed between A‐CD patients with and without clinical response, while MALT1 at W12 was lower in patients with clinical response than in those without clinical response (*p* = 0.008) (Figure 4D).

## 4 DISCUSSION

Although the original of MALT1 remains unclear, the current studies speculate that MALT1 might originate from mucosa‐associated lymphoid tissue.^24^ Additionally, MALT1, together with its caspase recruitment domain (CARD) family member BCL10, activates NF‐κB signaling pathway and regulates innate and adaptive immunoreaction.^25^, ^26^, ^27^ Furthermore, aberrant expression of MALT1 is linked with immunodeficiency.^28^, ^29^ In view of the fact that IBD patients are usually along with immunodeficiency; consequently, MALT1 may be dysregulated in IBD patients.^30^, ^31^ However, no relevant study investigates MALT1 expression in IBD patients. In this study, we found that MALT1 was elevated in IBD patients than HCs; additionally, MALT1 had good value to distinguish IBD patients from HCs as well as active patients from remission patients. The probable reasons might be that: (a) MALT1 was positively correlated with the accumulation of NF‐κB p65, while NF‐κB p65 was overexpressed in colitis mouse models.^24^, ^32^ Therefore, MALT1 was increased in IBD patients than HCs. (b) MALT1 promoted the differentiation of T cells into Th1 and Th17 cells via cleaving roquin and regnase‐1 proteins, which played crucial roles in IBD etiology.^33^, ^34^, ^35^ Hence, MALT1 could distinguish IBD patients from HCs and active patients from remission patients.

Several lines of evidence support that MALT1 regulates inflammation level in some autoimmune diseases, such as psoriasis, ankylosing spondylitis, rheumatoid arthritis, etc.^36^, ^37^, ^38^, ^39^ For instance, one study discloses that the inhibition of MALT1 suppresses the inflammatory response in proteoglycan‐induced ankylosing spondylitis mouse models.^37^ Considering that the autoimmune disease shared similar etiopathogenesis to some extent, meanwhile T‐cell activation and further leading to the production of various inflammatory cytokines commonly occurred in these patients, we hypothesized that MALT1 might be related to inflammation in IBD patients.^40^, ^41^, ^42^ In this study, we found that MALT1 was positively correlated with inflammation indexes and disease activity score of active IBD patients. The reason might be as follows: (a) MALT1 activated NF‐κB signaling pathway; meanwhile the activation of NF‐κB pathway was correlated with the secretion of various inflammatory cytokines (including IL‐1, IL‐6, TNF‐α, etc.) as well as the severity of intestinal inflammation.^43^, ^44^ Hence, MALT1 was positively associated with inflammation indexes in IBD patients. (b) MALT1 stimulated the activation of immune cells and promoted the disruption of intestinal endothelial cells through proteolytically cleaving cylindromatosis, while the disrupted gut homeostasis was thought to be linked with the severity of IBD symptoms.^45^, ^46^, ^47^ Thus, MALT1 was positively related to disease activity of IBD patients. (c) The dysregulation of MALT1 was positively correlated with microbial pathogens, which had been reported to be associated with the inflammatory status in IBD patients.^48^, ^49^, ^50^ Therefore, MALT1 was related to inflammation indexes in IBD patients.

Apart from what mentioned above, we also disclosed that MALT1 in both A‐CD and A‐UC patients was gradually declined after treatment. Of note, MALT1 at W4 and W12 in A‐UC patients as well as MALT1 at W12 in A‐CD patients associated with clinical response. The possible reasons might be that: (a) As described, MALT1 expression indicated the inflammation level in IBD patients, whose inflammation level was declined after treatment. Therefore, MALT1 was gradually decreased after treatment. (b) After achieving clinical response, the inflammation in IBD patients was greatly alleviated, then MALT1 expression decreased consequently.^51^ As a result, the rapid decline in MALT1 could disclose treatment response in IBD patients.

Some limitations occurred in this study. First, the number of patients who were enrolled in the study was relevantly small, which needed more samples to further valid the outcomings. Second, this study enrolled HCs to evaluate the value of MALT1 in assistance of diagnosing IBD, while we did not recruit non‐inflammatory intestinal disease patients as disease controls, which was necessary in the further study. Third, MALT1 level was only determined at the baseline, W4 and W12 after treatment, while the long‐term change of MALT1 level was not clear, which needed further validation. Fourth, the correlation of MALT1 with endoscopic remission was not assessed yet, which was needed in further study. Fifth, it might be an alternative and more visual way to determine MALT1 expression in feces, which needed to be investigated in the further study. Sixth, MALT1 and calprotectin might have a potential relationship which deserved further study. Seventh, the upstream pathway of MALT1 was still unclear, which needed to be explored in the future study.

## 5 CONCLUSIONS

In general, MALT1 is abnormal expressed and related to aggravated disease activity score, inflammation indexes as well as treatment response in IBD patients. Hence, MALT1 may serve as a potential biomarker for disease surveillance and treatment outcome prediction of IBD.

## CONFLICT OF INTEREST

No potential conflict of interest was reported by the authors.

## Data Availability

The datasets generated during and/or analyzed during the current study are available from the corresponding author on reasonable request.
